# Re‐Evaluating Hot Mitochondria: Too Slow to Cool

**DOI:** 10.1111/apha.70220

**Published:** 2026-04-17

**Authors:** Jason R. Treberg, Ryan J. Mailloux

**Affiliations:** ^1^ Department of Biological Sciences University of Manitoba Winnipeg Manitoba Canada; ^2^ School of Human Nutrition McGill University Quebec Canada; ^3^ Department of Biochemistry, Faculty of Medicine and Health Sciences McGill University Montreal Quebec Canada

**Keywords:** metabolism, mitochondria, substrate oxidation, temperature, thermodynamics

## Abstract

**Aim:**

Mito Thermo Yellow (MTY) is a mitochondrially targeted fluorophore that shows marked fluorescence quenching with increasing temperature, allowing for interrogating temperature dynamics in the mitochondria of live cells. Here we re‐evaluate published MTY fluorescence responses used to argue in favor of the ‘hot mitochondria’ concept; the assertion that mitochondria operate while maintaining substantial (> 10°C) apparent temperature gradients (Δ*T*_*app*_) between themselves and their cellular environment.

**Results:**

We find that MTY fluorescence kinetics are incompatible with the expected dynamics of mitochondrial heat production and diffusion. We further explore the published effects of mitochondrial inhibitors on MTY, and related evidence for Δ*T*_app_ of > 10°C, again concluding results are inconsistent with the expected heat production dynamics. Thus, assertions of Δ*T*_app_ > 10°C between mitochondria and their cellular environment based on MTY fluorescence intensity changes are unlikely to be reporting a signal that is uniquely intramitochondrial temperature. In addition to these analyses, we further argue that the inference mitochondria can operate at an internal temperature of > 48°C, as reported using MTY, is improbable as these internal temperatures would cause protein denaturation and aggregation and induction of the heat shock (HSR), unfolded protein (UPR), and integrated (ISR) stress responses.

**Conclusion:**

Taken as a whole, we conclude MTY and similar tools must be re‐evaluated in regard to if they are providing solely information on local temperature and thus are so far inadequate, unto themselves, to demonstrate the existence of hot mitochondria.

## 1 Introduction

Temperature is a measure of the average internal kinetic energy of the particles that make up a system's matter, while heat can be seen as the form of energy that can transit through matter. In this context, heat is expected to be dissipated by conductance down temperature gradients. Heat influences the rate of reactions that living systems rely on, making temperature a ubiquitous factor that affects life on our planet. In most Eukaryotic cells, the bulk of heat liberated during metabolism occurs from substrate oxidation within the mitochondrion fueling oxidative phosphorylation. Importantly, during oxidative phosphorylation, some potential energy is conserved within the bonds of the ATP generated in the mitochondrial matrix and that potential heat is exported from the mitochondrion to the cytosol where much of it is liberated during cellular work (reviewed in [1]). All living organisms produce heat, regardless of their approach to thermoregulation, be it ectothermy or endothermy; however, in mammalian cells it has been argued that mitochondria maintain a thermal gradient of over 10°C between their inner compartment (the matrix space) and the surrounding cytosolic environment [2]. This claim of such a remarkable apparent temperature gradient (Δ*T*_*app*_) across mitochondria was based on use of an intramitochondrial probe, Mito Thermo Yellow (MTY) which displays fluorescence quenching as a function of temperature [3]. Within cells, MTY could act as an intramitochondrial thermometer because it is both targeted and accumulated within the mitochondrion by the mitochondrial membrane potential (ΔΨ_M_) [3]. The plausibility and physiological consequences of such a substantial Δ*T*_app_ have been discussed in several reviews, some accepting the idea, or even building off it, while others range from ambivalence to rejecting it outright [4, 5, 6, 7, 8] (as a non‐comprehensive list). Importantly, more recent support for an extreme mitochondrial Δ*T*_app_ has included demonstrating similar patterns in additional mammalian cell types and expanding the types of cell demonstrating an Δ*T*_app_ of circa 10°C to include an ectothermic species [9]. Importantly, in the same study, an alternative fluorescent probe that uses quenching of a ratiometric fluorescent signal (mito‐gTEMP), rather than MTY's simple intensity‐based response, has been used as further support of these extreme values for Δ*T*_app_ of 10°C or more [9]. Together, these data [2, 9] have been argued [10] to indicate a Δ*T*_app_ of ≥ 10°C in mitochondria, a concept we will refer to as ‘hot mitochondria’. To emphasize, the possibility of smaller and potentially transient mitochondrial Δ*T*_app_ upon chemical uncoupling are supported by diverse experimental evidence, using both intact cells [as examples, see [11, 12]] and isolated mitochondria [13], which we do not contest; however, the hot mitochondria hypothesis argues mitochondria normally function with a sustained and extreme thermal gradient (≥ 10°C) between themselves and the surrounding cell, which does warrant further scrutiny.

Here we assess several outstanding aspects in need of clarifying prior to accepting the hot mitochondrial concept, using a bioenergetics perspective to interrogate if mitochondria likely operate ’hot’ (> 50°C in mammalian cells) or not. Prior to addressing the experimental studies relating to the hot mitochondria hypothesis, it is important to put mitochondrial respiratory control, the partitioning of cellular heat production and the mitochondrial handling of MTY in context. We then examine past theoretical arguments on how cellular power output is too low for generating substantial temperature gradients, known as the 10^5^ power gap [4, 7, 14]. Finally, we illustrate that the slow cooling and response to classic mitochondrial inhibitors are both incompatible with the MTY‐signal being reflective of mitochondrial heat flux; thus, this signal is inconsistent with being uniquely reflective of intramitochondrial temperature.

## 2 Mitochondrial Heat Production

Putting our arguments in context requires addressing heat production in relation to mitochondrial function and respiratory fluxes. The release of heat is expected from respiring mitochondria because the oxidation of substrates along with the reduction of oxygen to water is both favorably, exergonic (Δ*G* < 0), and exothermic (Δ*H* < 0). Unlike the concepts behind a mitochondrial Δ*T*_*app*_, heat production by mitochondria is widely acknowledged as a product of the biochemical and biophysical energy transformations that contribute to mitochondrial electron transport and the chemiosmotic coupling of oxidative phosphorylation (OXPHOS) to maintain the capacity for cellular work via the phosphorylation potential [15]. Microcalorimetry has been combined with indirect calorimetry (respiratory oxygen flux) to show that when mitochondria oxidize substrates, the apparent Δ*H* (measured in J per atomic‐O consumed) varies depending on factors including the respiratory state and coupling efficiency of the preparation [16, 17]. The Δ*H* per O consumed is measurably higher for mitochondria in a non‐phosphorylating (‘leak’) state than when oxygen flux is elevated by addition of ADP to allow F_0_F_1_ATPase flux and engage OXPHOS [17]. Mitochondria in a leak‐state that are from poorly coupled preparations have a higher Δ*H* (in J O^−1^) than well coupled ones. Similarly, when substrate oxidation by liver mitochondria is activated by chemical uncouplers, the Δ*H* was in general agreement with the expected enthalpy of combustion of the substrates used [17]. Thus, unsurprisingly, the bulk of mitochondrial thermogenesis is a product of respiratory substrate oxidation. As shown elsewhere [18], the rate of heat production by mitochondria is expected to be directly proportional to the electron transport system (ETS) flux, but we must clarify that this is only directly true when uncoupled. An important distinction for our purposes is that while total cellular heat flux will be proportional to mitochondrial ETS flux and ATP turnover, the intramitochondrial heat production per pair of electrons through the ETS is lower during OXPHOS than at the same ETS flux when uncoupled (illustrated in the next section). This lower Δ*H* per O consumed during oxidative phosphorylation is to be expected since some of the energy is ‘trapped’ by the bond formation during re‐phosphorylation of ADP to ATP (reviewed in [1]). Therefore, in general, mitochondrial thermogenesis is primarily determined by the rate of substrate oxidation, even though the Δ*H* per O consumed may vary by as much as 50% between coupled and uncoupled respiration and much of the potential intramitochondrial heat is exported from the mitochondrion, within ATP during OXPHOS [17]. To emphasize, in terms of heat budgets, the net heat production at the cellular level will be comparable between OXPHOS and uncoupled mitochondria, but the site of heat release will vary because cellular demand for extramitochondrial ATP is also a major source of cellular free energy release. In regard to extramitochondrial heat formed per ATP used, this will vary substantially depending of the reactions involved. The Δ*G* of ATP hydrolysis under ‘cellular’ conditions is approximately −50–60 kJ mole^−1^ [15] while for phosphor‐transfers (such as protein phosphorylation or glucose‐6‐phosphate hydrolysis by glucose‐6‐phosphatase) is much lower (approximately −13 kJ mole^−1^).

## 3 Mitochondrial Respiratory Control: Using the Protonmotive Force as an Intermediate

**FIGURE 1:**
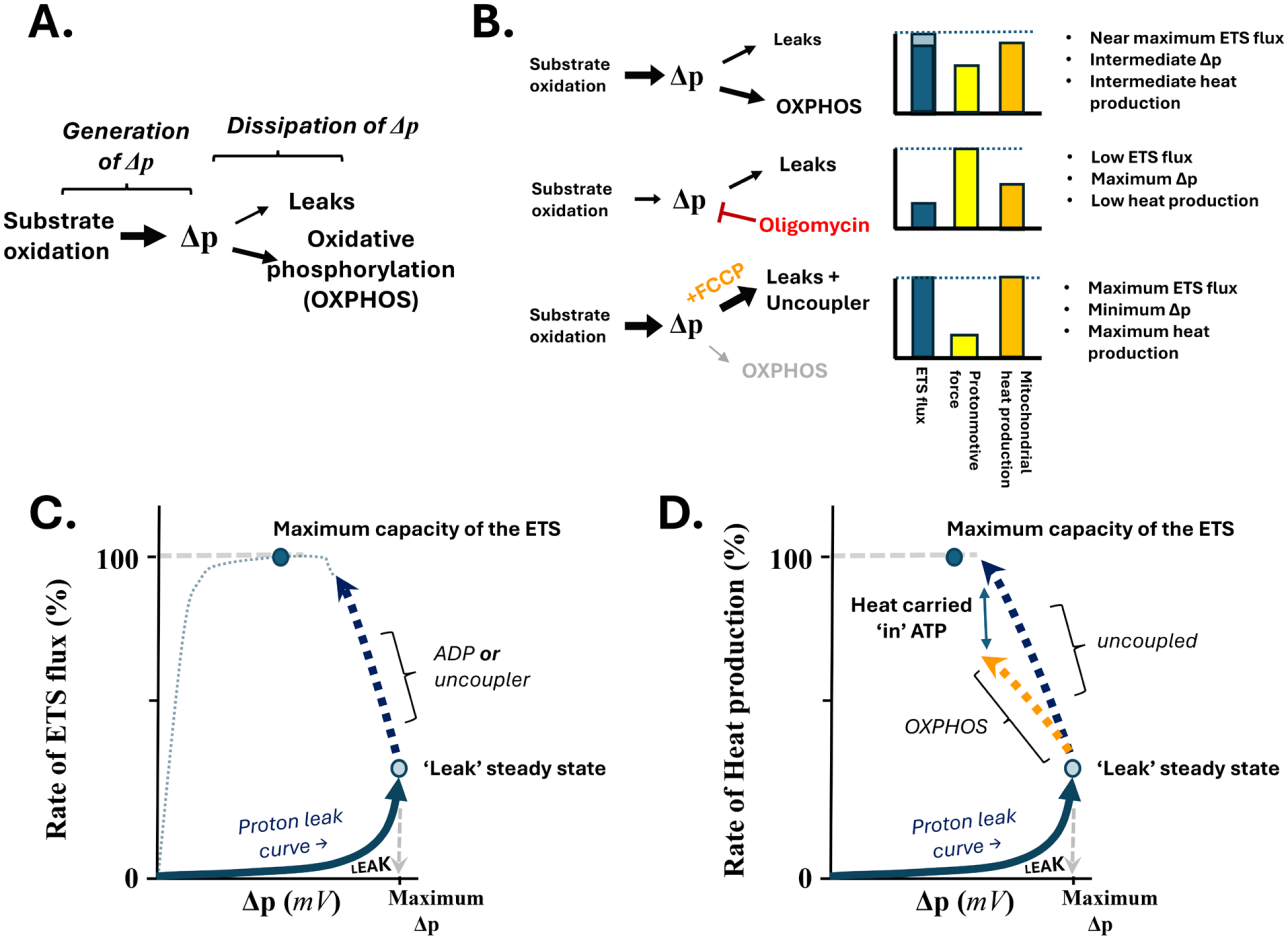
Mitochondrial respiratory control and intramitochondrial partitioning of heat production between coupled and uncoupled respiration. (A) Separation of mitochondrial electron transport and proton fluxes into systems generating and dissipating the protonmotive force (Δ*p*), where the Δ*p* is envisioned as an intermediate between substrate oxidation that generates the Δ*p*, and liberates heat within the mitochondrion, and the proton fluxes that dissipate Δ*p* (leaks and OXPHOS). (B) The relative responses of mitochondrial electron transport system (ETS) flux rate (blue), the protonmotive force (protonmotive force (Δ*p*)) (yellow) and mitochondrial heat production (orange) under three steady state conditions: Oxidative phosphorylation (OXPHOS) with concomitant modest physiologically relevant contribution via leak pathways (top), cells where OXPHOS is blocked by addition of oligomycin (middle) and cells where mitochondrial ETS flux has been maximized with an uncoupler (bottom). Note, mitochondria from some cells have the capacity to approach or reach maximal ETS flux via OXPHOS, with saturating phosphate and ADP, which is illustrated by the lighter blue bar in the top example; however the mitochondrial heat production would still be lower than the uncoupled system (bottom) as explained in the text around ATP as a carrier of potential heat. (C) Illustration of respiratory (ETS) flux control in relation to intrinsic Leaks (Proton Leak Curve) and the effect of adding uncoupler, or activation of OXPHOS, from a maximal ‘leak’ state with saturating respiratory substrate. (D) Illustration of the partitioning of heat flux from mitochondria emphasizing the expected decrease during OXPHOS relative to the uncoupled state at a comparable rate of ETS flux. Synthesis based on [1, 15, 19, 20].

Mitochondrial respiration includes both electron transport and proton/charge translocation to generate the protonmotive force (Δ*p*), which is predominantly membrane potential (ΔΨ_M_) and to a lesser degree the pH gradient (ΔpH). Substrate oxidation drives the electron transport system (ETS), generating the Δ*p* while (i) passive (basal) proton leak, (ii) induced (chemical or uncoupling proteins) leak and (iii) oxidative phosphorylation (OXPHOS) driving F_0_F_1_ATPase influx of free protons all contribute to the dissipation of the Δ*p* (Figure 1A). The Δ*p* can be illustrated as an intermediate between the substrate oxidation that generates it along with coupled (OXPHOS) and uncoupled dissipation of the Δ*p* (Figure 1A).

While not necessarily a consensus view of respiratory control [21], this model is a useful heuristic for our purposes. Within this conceptualization of respiratory control, there will be an underlying proton leak curve, which is typically measured by titrating the substrate oxidation capacity while the mitochondria respire in a ‘non‐phosphorylation’ (or leak) state. The leak curve illustrates the rate of proton leak as the Δ*p* varies, assuming the rate of proton translocation equals the rate of proton leak at a given Δ*p*. The leak rate follows a curving ‘non‐Ohmic’ shape, indicating how the rate of electron transport changes with the Δ*p* when mitochondria are in a leak state–state (non‐OXPHOS respiratory state without exogenous uncoupler). In this model, when in a leak‐state, the ETS flux is primarily controlled by the intrinsic permeability of the inner mitochondrial membrane to proton influx and the substrate oxidation capacity of the system [15]. Considering different cellular steady state conditions, including (i) a ‘routine’ rate of cellular metabolism, (ii) a pharmacologically clamped leak state (such as cells treated with oligomycin) and (iii) a maximum rate of ETS flux using a chemical uncoupler, straightforward expectations for the ETS flux, Δ*p* and rate of mitochondrial heat generation can be expected (Figure 1B). To conceptualize how these are expected, it is best to picture a 2‐dimensional system where the Δ*p* intermediate is the independent variable and the rate of ETS flux is the dependent axis (Figure 1C). From the maximum steady‐state ‘leak’ rate for a given capacity of substrate oxidation, if the Δ*p* is dissipated by either activation of forward F_0_F_1_ATPase flux via OXPHOS or addition of uncouplers, the rate of electron transport will elevate while the steady‐state Δ*p* will shift to a lower value than when in the leak state (Figure 1C). However, in terms of intramitochondrial heat liberation, during OXPHOS the net export of ATP from the mitochondrion is expected to cause less intramitochondrial heat production than if the same level of oxygen flux was achieved by addition of uncoupler (Figure 1D).

While the concepts behind this approach to respiratory control were initially developed with isolated mitochondria, conceptually and experimentally these tools translate well to ΔΨ_M_ estimates of Δ*p* combined with oxygen flux rates with intact cells [19, 20] and perfused liver or muscle [22, 23], making this approach valuable for our purpose of explaining the influence of pharmacological manipulations on mitochondrial energy transformation in cells. To that end, there have been at least two explanations on how mitochondrial F_0_F_1_ATPase flux may directly contribute to the claimed Δ*T*_*app*_, one based on the idea that molecular turbulence in the vicinity of the matrix facing side of F_0_F_1_ATPase might register as temperature with MTY on an extremely small spatial scale [4] and the other a detailed description of how F_0_F_1_ATPase could act as a heat engine hypothesized to generate the Δ*T*_app_ [7]. Both cases are worthy of further investigation but, also in both cases, the proposed mechanisms are unlikely to contribute support for a hot mitochondria hypothesis. This is because uncouplers increase mitochondrial heat production while also decreasing the *F*_0_ *F*_1_ATPase influx at a given Δ*p*. Therefore, in both cases, if F_0_F_1_ATPase was specifically important to the thermal gradient, then adding uncouplers to cells should appear to decrease the mitochondrial Δ*T*_*app*_.

## 4 The Fate of MTY in and Around Mitochondria Is ΔΨ_M_ ‐Dependent

**FIGURE 2:**
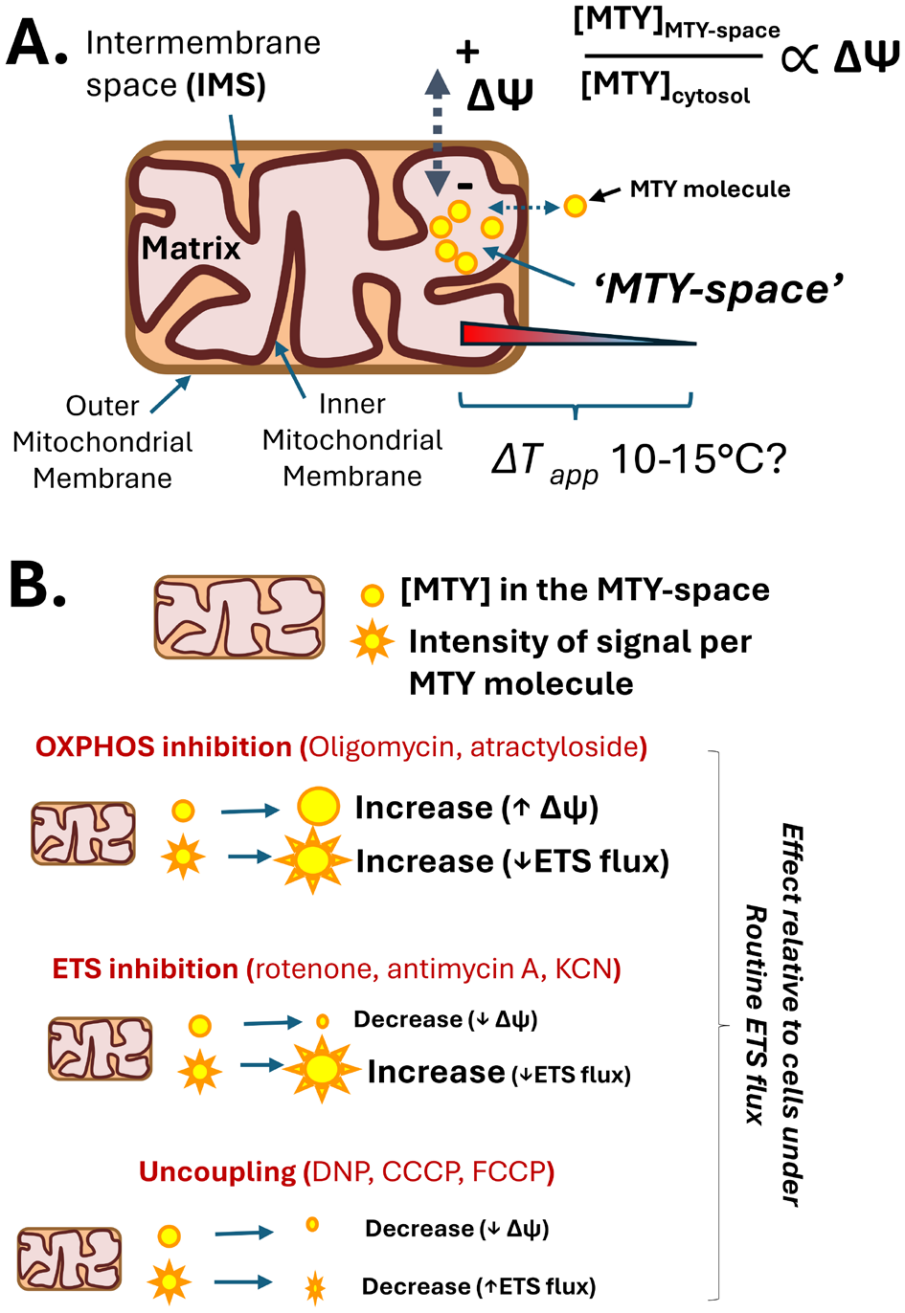
The influence of bioenergetics on the MTY fluorescence in mitochondria. (A) compartmentalization of mitochondria and membrane potential (Δψ) dependence of MTY uptake into the MTY‐space within mitochondria. (B) Anticipated effect of classic mitochondrial inhibitors, or uncouplers, on intramitochondrial levels (yellow circle size) and response of electron transport system (ETS) flux on overall MTY response. Note, MTY fluorescence increases with relative ‘cooling’ [3].

Mito Thermo Yellow is accumulated within the mitochondrion in a membrane potential (ΔΨ_M_) dependent manner (Figure 2A) and its fluorescence responds to extracellular changes in temperature very rapidly (within seconds) [3]. Like many other lipophilic compounds that are driven into mitochondria by the ΔΨ_M_, where exactly the MTY accumulates and how much is free, bound to macromolecules or forming aggregations within the mitochondrial matrix is not fully known, although some MTY is known to bind to aldehyde dehydrogenase‐2 [24]. For this reason, we refer to the MTY‐space as where the intramitochondrial response of MTY fluorescence is taking place; however, the ΔΨ_M_ dependent uptake indicates that the bulk of the MTY preloaded within a cell will be localized in the mitochondrial matrix regardless of being free or bound. Once concentrated within the mitochondrion, the effective capacity of MTY to act as a thermometer would be on the nanometer scale. Such small spatial scales lead to an expectation of extraordinarily rapid equilibration of heat from within the MTY‐space to the cellular environment due to diffusive heat exchange, as is confirmed with extracellular manipulation of temperatures [3]. Given the well‐established expectations of classic mitochondrial pharmacological manipulations (such as oligomycin or uncouplers), and MTY ΔΨ_M_ dependent uptake into mitochondria, it is clear how effectors that may influence the ΔΨ_M_ may indirectly manipulate the intramitochondrial concentration of MTY (Figure 2B), making this a challenging signal to calibrate above and beyond the additional chemical influences (such as pH and ionic strength) on MTY's fluorescence, as noted elsewhere [25, 26].

## 5 The Apparent Cooling of ‘Hot Mitochondria’

**FIGURE 3:**
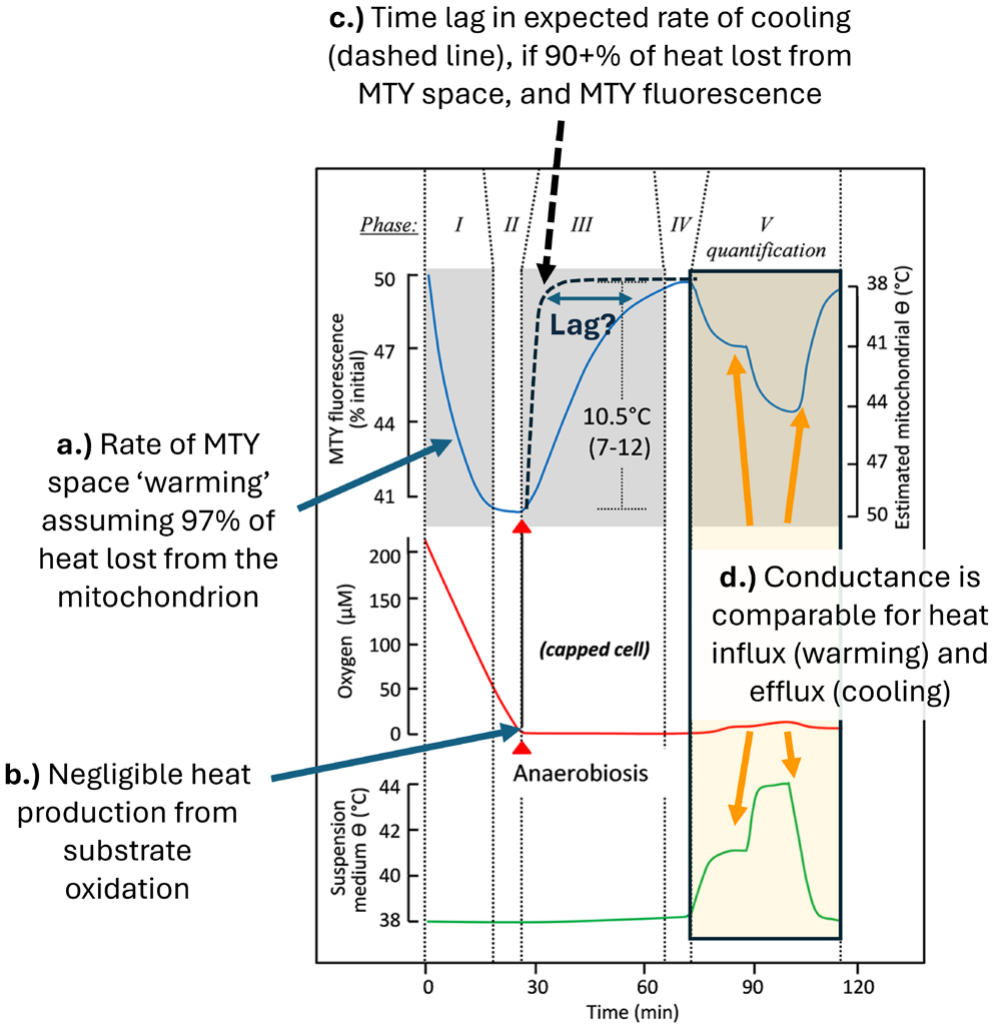
Responses of Mito Thermo yellow (MTY) fluorescence are inconsistent with expected kinetics of heat exchange. The MTY fluorescence response while cells are actively respiring (Phase I), have reached a stable steady‐state MTY fluorescence as the same respiration rate (Phase II), followed by apparent cooling from cessation of heat generation due to anoxia (Phase III) and new steady‐state fluorescence of non‐respiring mitochondria (Phase IV), followed by calibration of the fluorescence by altering the temperature of the medium by changing the set water bath temperature (Phase V). While the apparent rate of warming (A) can be explained based on heat efflux, on reaching anoxia (B) the apparent rate of cooling based on the MTY fluorescence lags behind the dashed expected rate (C) that would be required to justify the slow rate of warming in Phase I. During the calibration phase there is no evidence that mitochondria are a significant barrier to heat exchange by influx or efflux from the MTY space (D). Figure is modified Figure 1C from [2].

In Chrétien et al.'s [2] initial study using MTY to argue the case that mammalian mitochondria operate near or above 50°C, raw traces were provided to illustrate experimental results (e.g., see Figure 3 which is a subset of Figure 1 from [2]). The apparent warming of the MTY‐space in respiring cells is slow, taking > 15 min to reach a steady‐state, while O_2_ flux proceeds at a relatively constant rate (Figure 3A). The authors explain how the slow warming of the MTY‐space is consistent with a stable rate of heat production combined with most of the heat produced being lost from the mitochondrion (calculations can be found within the Supporting Information of [2]), which is sound. However, the cells are in a sealed cuvette so they ultimately consume the O_2_ to apparent functional anoxia (Figure 3B) at which point heat production from mitochondrial substrate oxidation is expected to decline substantially if not approach zero. If most of the heat formed during the warming phase is being lost from the MTY‐space, then dissipative cooling should cause the MTY‐space to cool rapidly under anoxia (dashed line, Figure 3C); however, contrary to expectation, the MTY‐space appears to cool at a remarkably slow pace, taking about 30 min in this specific case to dissipate a Δ*T*_app_ of roughly 10°C [2]. As the authors correctly note, conductance of heat out of the mitochondrion could explain the extended time required for the Δ*T*_app_ of the MTY‐space to stabilize above the assay medium. But the extreme lag in the MTY response after heat production should have dropped markedly indicates either the MTY response is not uniquely a strict measure of MTY‐space temperature, or there must be a marked change in heat conductance between respiring and non‐respiring mitochondria. The calibration steps (Figure 3D) clearly dispute the possibility of major directional differences in heat conductance capacity between the bathing medium and the MTY‐space. To emphasize the theoretical magnitude of the problem slow cooling presents to the hot mitochondria hypothesis, we must first address if significant thermal gradients within cells can be generated; also known as the metabolic power gap.

## 6 Is the 10^5^ Power Gap a Major Theoretical Barrier Against the Case for Hot Mitochondria?

As noted in other cases [4, 6, 7, 14, 27], the theoretical arguments made [14] on how cells typically have such low power output that they should be incapable of maintaining temperature gradients of more than 10^−5^ K (or °C) are a major conceptual challenge to the hot mitochondria hypothesis; often referred to as the 10^5^ gap. The rationale is based on the following:

ΔT=P/κ*L

where Δ*T* indicates the temperature gradient (in K or °C), P is the power output (W), *κ* is the thermal conductivity coefficient (W m^−1^ K^−1^) and L is the distance the heat must travel through (m). In the original work [14], it is recognized that for mitochondria within cells this could be seen more as a 10^−4^ gap, and as raised elsewhere [7] the value for *κ* could be much lower in cells (~0.11, see [28]) than expected from dilute aqueous solutions where *κ* is ~0.6. The original work on this power gap [14] used a value of 1.0, but using a more intracellular relevant value brings the mitochondrial gap towards 10^−3^. Moreover, as already noted elsewhere [4], limiting cellular power to 100 pW per cell may be unrealistic, for example in hard‐working mammalian cells in vivo like cardiomyocytes. Direct calorimetry of permeabilized guinea pig right ventricle bundles indicates the mitochondria within can produce heat at ~64 mW cm^−3^ when uncoupled [29]. Assuming guinea pig cardiomyocytes have a similar volume to adult rat right ventricle of ~21 600 μm^3^ [30], or 4.6 × 10^7^ cells cm^−3^, this gives 1390 pW cell^−1^. These heat outputs for permeabilized cardiac tissue are in reasonable agreement with isolated perfused guinea pig heart oxygen flux rates, circa 0.18 mL O_2_ g^−1^ min^−1^, that predict heat production of ~62 mW g^−1^ [31] assuming aerobic metabolism is fueled by complete glucose oxidation (~230 kJ per mole O consumed). Thus, the traditional 10^5^ gap argument [14] is not necessarily as insurmountable a theoretical barrier to the hot mitochondria hypothesis as sometimes considered, potentially being a 10^2^ gap or less for mitochondria in high energy demand cells like cardiomyocytes. The rationale behind the 10^5^ gap argument does provide a clear challenge to the slow cooling rates in the MTY data used to support the contention mitochondria normally operate at a markedly higher internal temperature than their surroundings [2, 9].

## 7 Intramitochondrial Cooling: The Theoretical Gap Hot Mitochondria Cannot Span

While the in vivo mitochondrial structure is complex, making estimates for net heat flux per unit surface area challenging, by simplifying to the linear distance heat must travel to escape the MTY‐space we can apply similar rationale used to derive the 10^5^ gap problem to estimate the heat flux per unit distance using:

q=−κ*ΔT/L

where *q* is the heat flux (in W) and the other terms are as already defined above. If we assume that there was a ~10 K thermal gradient between the MTY‐space and the cytosol when the system goes anoxic (Figure 3), and use a rough estimate of 500 nm as the diffusion distance to the extramitochondrial space [14], then with a range of *κ* from ~0.1 to 1 W m^−1^ s^−1^ the initial rate of heat dissipation on reaching anoxia would be ~2–20 × 10^6^ J s^−1^. Assuming mitochondria are in the range of 500 nm or less across, then the heat dissipating from the MTY‐space is leaving volumes of less than a microlitre in scale. Moreover, a 10°C (10 K) thermal gradient has only about 4 × 10^−2^ J of excess heat per microlitre of aqueous medium. While some delay or lag is expected for pharmacological inhibitors to reach their targets and take effect, or even for the transition from aerobic to anoxic conditions, the small diffusion distance out of the MTY‐space implies there should be approaching zero lag in heat dissipation as ETS flux changes. However, several minutes, and often 10's of minutes, are required to reach the new apparent thermal equilibrium [2, 9], long after the mitochondrial ETS flux should have stabilized at a new steady state. This suggests the true theoretical gap for hot mitochondria is not in establishing thermal gradients, which may be experimentally demonstrated by pharmacological elevation of mitochondrial heat output within cells [11, 12, 32] and isolated mitochondria [13]; rather, it is the inexplicably slow dissipation of the Δ*T*_app_ that makes the experiments in support of the hot mitochondria hypothesis incompatible with Fourier's law of heat conductance (Figure 3).

## 8 Alternative Explanations for the Lag?

The lagging heat dissipation does not appear to be explainable by diffusion barriers to heat conductance (Figure 3D); however, there are possible intramitochondrial sources of heat in the experiment that should also be addressed: (i) leaks in the experimental system allowing back diffusion of atmospheric oxygen into the respirometry cell and (ii) the reversal of F_0_F_1_ATPase. Regarding the back diffusion of oxygen into the chamber, this would likely lead to a condition where complex IV has near, or complete, control over ETS flux due to oxygen availability leading to kinetic limitation on substrate oxidation. In the representative experiment (Figure 3), the cells are initially consuming oxygen at about 120 pmol O_2_ s^−1^, assuming 28 min to apparent functional anoxia. While we have not used the specific cuvette the authors describe [2], in our hands, oxygen back‐diffusion into closed cell respirometers with capillary ports for additions via syringe, like that described, are ~ an order magnitude lower than the unencumbered rate of oxygen consumption we estimate above for the HEK293 cells prior to reaching anoxia (Figure 3). Thus, even though the rate would be low, a plausible intramitochondrial heat production could hypothetically be occurring due to atmospheric oxygen input via leaks in the respirometry cell. But even if there is demonstrable atmospheric oxygen influx, the change in heat production rate would be expected to occur as a rapid change in steady‐state heat output, not a slow gradual dissipation of ETS flux; therefore this seems unlikely to explain the slow cooling of apparently hot mitochondria.

**FIGURE 4:**
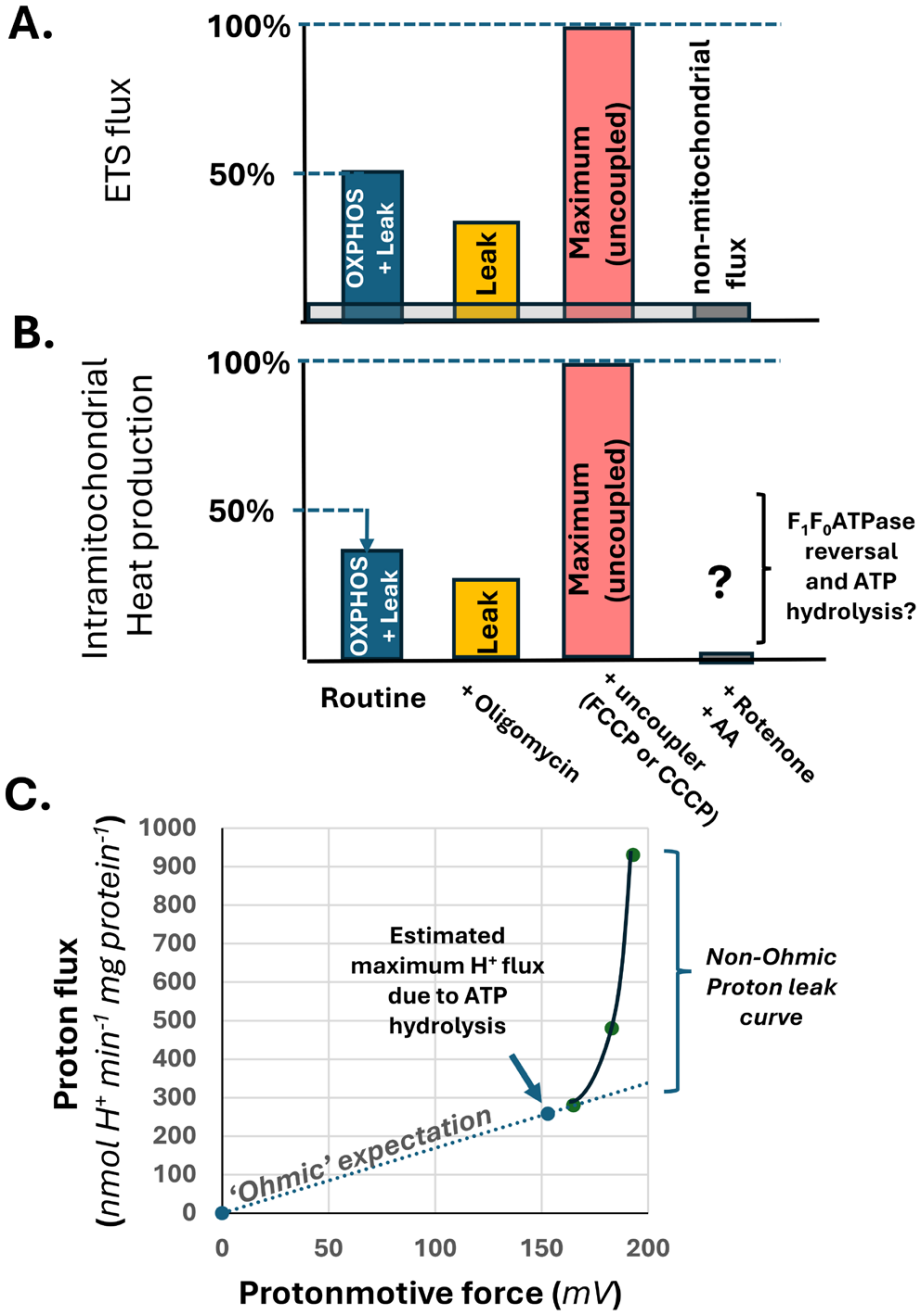
Schematic of general expectation of ETS and heat fluxes during cellular energetics experiments in relation to estimation of ATP hydrolysis drive proton flux by isolated rat skeletal muscle mitochondria at 37°C. (A) Typical relative expected results of different steady states during cellular energetic experiment where the ‘routine’ rate of oxygen flux is used to measure ETS flux made up of both leak and OXPHOS driven respiration. Pharmacological clamping into a leak respiratory state (with oligomycin) lowers both ETS flux and heat production while chemical uncoupling to measure maximum ETS flux maximizes heat production. ETS inhibitors like rotenone or antimycin A (AA) are generally used to correct for non‐mitochondrial oxygen consumption. (B) the ETS fluxes converted to approximate intramitochondrial heat production under the same hypothetical conditions; Note, that ETS flux (A., upper panel) and intramitochondrial heat production (B., lower panel) are qualitatively similar but have differing quantitative responses in part due to non‐mitochondrial oxygen consumption and transport of heat equivalents in ATP out of the mitochondrion. (C) Rates of oxygen consumption by isolated rat muscle mitochondria (from [38, 39]) in a non‐phosphorylating (leak) state respiring on either malate, glutamate plus malate or succinate in the presence of rotenone are used to plot the non‐Ohmic proton leak curve. Oxygen fluxes were converted to steady state H^+^ flux assuming 10, 10 or 6 proton equivalents extruded per atomic O consumed from malate, glutamate and malate or succinate and rotenone respectively. The Ohmic line is extrapolated from zero to the malate respiration value, assuming this is approaching the end of the non‐Ohmic relationship of the leak curve. The estimated rate of proton flux is from the Ohmic relationship and the protonmotive force that can be maintained by ATP hydrolysis [34] and is used to calculate the rate of ATP hydrolysis assuming 11 H^+^ translocated per 3 ATP (one turnover of the ATPase and concomitant phosphate carrier exchange). See text in **Alternative explanations for the lag?** section for further details.

In the case of F_0_F_1_ATPase, it is important to consider an alternative possibility where heat liberation from cytosolic ATP, generated by glucose fermentation to lactate, is scavenged by mitochondria to maintain the Δ*p*. Importantly, if the intramitochondrial ATP hydrolysis is sufficiently high, this could contribute to some Δ*T*_app_ when the ETS is blocked, and the process would cease with oligomycin, or adenine nucleotide translocase inhibitors like atractyloside or bongkrekic acid. The hydrolysis of accumulated ATP once mitochondria have consumed the oxygen in a closed cell leads to measurable heat flux of about −29 kJ per mole ADP formed [17], which was similar to a measured change in enthalpy of 35.8 kJ mole^−1^ of ATP formed in another study [16]. However, those studies used mitochondrial preparations form liver, where the phosphorylating system may not be able to maximize ETS flux as seen with skeletal muscle preparations where the phosphorylation system can attain comparable rates of ETS flux (at supraphysiological levels of phosphate and ADP) as seen with chemical uncouplers [33]. To address an upper limit of how much heat the F_0_F_1_ATPase might contribute, we sought a means to derive how much proton flux could be generated by maintaining the Δ*p* via ATP hydrolysis. The Δ*p* that isolated rat skeletal muscle mitochondria can maintain via ATP hydrolysis has been measured [34] so we collected other data from the literature using the same apparatus and similar assays to recreate the typical ‘non‐Ohmic’ pattern seen with proton leak curves [35, 36], which notably also occurs with mitochondria in situ with intact cells [37]. The generally expected relative responses for the ETS flux with intact cells transitioning from a routine state (which has both a leak and OXPHOS component) to a pharmacologically clamped leak‐state would be roughly a 50% decreased in mitochondrial oxygen flux. The routine rate of ETS flux would be substantially lower than the expected uncoupled maximum ETS flux rate (Figure 4A). By converting the ETS flux rates to anticipated intramitochondrial heat liberation, the leak‐state will be expected to have lower mitochondrial heat production than the routine state, even accounting for the lower heat released per O consumed from ETS flux due ATP export during OXPHOS (Figure 4B). These relative responses give the framework to then ask if F_0_F_1_ATPase can generate more heat than mitochondria respiring in a leak‐state.

Specifically, we collected data on skeletal muscle mitochondrial isolated from female Wistar rats using the similar methodology to measure Δ*p* by uptake of methyltriphenylphosphonium [34, 38, 39]. Under these conditions, isolated mitochondria hydrolyzing ATP maintain a Δ*p* ranging from −141 to −166 mV in the presence of ETS inhibitors including rotenone, antimycin A or CN^−^ [34]. Using the same technique on the same rat strain to measure the Δ*p* under non‐phosphorylating (‘leak’) respiration (Figure 4C) produces a reasonable replication of the expected non‐Ohmic proton leak curve [40]. If we assert that the non‐Ohmic relationship only occurs at a Δ*p* greater than the lowest measured value on the respiration driven leak curve, −165 mV with malate as the only substrate, then a simple linear interpolation can estimate the maximum rates of proton pumping required to maintain the Δ*p* over the range expected from ATP hydrolysis (Figure 4A). Mammalian F_0_F_1_ATPase has 8 c‐subunits [41], therefore, hydrolysis of 3 ATP in the matrix will be equivalent to expulsion of 11 H^+^ (8 by the ATPase itself, 3 accompanying the net inorganic phosphate efflux to the extramitochondrial space). Using a median of −153 mV, the rate of proton translocation expected to maintain this Δ*p* against the passive leaks is estimated to be ~260 nmol H^+^ min^−1^ mg protein^−1^. The standard enthalpy of ATP hydrolysis is about −19.7 kJ mol^−1^ [42, 43]. However, temperature, ionic strength along with pH and pMg all influence the thermodynamics of ATP hydrolysis [44]. For simplicity we use the highest estimate for ATP hydrolysis, −28.7 kJ mol^−1^, at a physiologically relevant ionic strength of 0.25, that accounts for pH and magnesium at a temperature of 37°C as reported elsewhere [44]. Using this higher value of Δ*H* provides an upper estimate of heat liberation to maintain a Δ*p* of −153 mV solely by ATP hydrolysis driven F_0_F_1_ATPase reversal can be determined based on:

$$\begin{array}{c} \text{Maximum rate heat release}=\text{Rate of}\;H\\ \;+\text{extrusion}\times \left(\mathrm{ATP}/H+\text{extruded}\right)\times {\mathrm{\Delta H}}_{\mathrm{ATP}\;\text{hydrolysis}} \end{array}$$

$$=260\;\text{nmol}\;H^{+}\;\min^{-1}\;\mathrm{mg}\;{\text{protein}}^{-1}\times \left(3\;\mathrm{ATP}/11\;H^{+}\right)\times –28.7\;\mathrm{kJ}\;{\mathrm{mol}}_{\mathrm{ATP}}$$

$$=2.04\times 10^{-3}\;J\;\text{heat release}\;\min^{-1}\;\mathrm{mg}\;{\text{protein}}^{-1}$$

The rates of oxygen flux that were used for the non‐Ohmic leak curve expressed as proton flux rates (Figure 4C) can be converted to heat production, in J min^−1^ mg protein^−1^, using Δ*H* values for NADH oxidation (−260 kJ mole^−1^) or succinate oxidation to fumarate (−150 kJ mole^−1^) with uncoupled mitochondrial preparations [45]; giving leak state heat fluxes of 7.3 × 10^−3^ J min^−1^ mg protein^−1^ to 23.3 × 10^−3^ J min^−1^ mg protein^−1^ with malate and succinate oxidation respectively. Even if these values were further corrected for the approximately 46%–48% lower heat flux with mitochondria in a leak‐state relative to the enthalpy per O with uncoupled ETS flux [17], these results clearly indicate ATP hydrolysis during anoxia seems incapable of generating the level of heat production seen with mitochondria in a leak‐state, and thus should produce much less heat than cells in a routine state of respiratory flux. Therefore, ATP hydrolysis appears incapable of explaining the large mitochondrial Δ*T*_*app*_ reported using MTY, let alone the 50°C or more alleged for mammalian cells [2, 9].

## 9 Interrogation of Respiratory Inhibitors on Mitochondrial Δ*T*_app_ Leads to More Questions Than Answers

With MTY responses not fitting the expected kinetics of heat generation and dissipation in mitochondria, we turn to how well the reported effects of mitochondrial inhibitors fit the expected dynamics of heat production. If the bulk of mitochondrial thermogenesis is due to substrate oxidation, then well‐established inhibitors of specific mitochondrial processes can be valuable tools. Well‐established mitochondrial inhibitors and uncouplers can test for expected relationships between thermodynamically relevant thermogenesis and the generation of a mitochondrial Δ*T*_app_ monitored with MTY or other candidate nanothermometers like mito‐gTEMP. Indeed, one of the more compelling experimental supports for mitochondrial Δ*T*_app_ is that uncoupling markedly elevates ETS flux and appears to enhance Δ*T*_app_, while inhibition of respiratory flux abolishes the Δ*T*_app_ [2]. More recently, using both MTY and the ratiometric alternative mito‐gTEMP, mitochondrial Δ*T*_app_ was demonstrated to be more sensitive to inhibition of F_0_F_1_ATPase with oligomycin than to blockage of electron transport at the level of complex I, III, or IV [9]. Oligomycin having the greatest dissipation of Δ*T*_app_ is puzzling because oligomycin does not prevent substrate oxidation per se, but simply removes the capacity of F_0_F_1_ATPase to elevate oxygen flux via proton return during oxidative phosphorylation. Therefore, in general, oligomycin is much less capable of inhibiting substrate oxidation, measured as oxygen flux, than ETS inhibitors like KCN, rotenone, or antimycin A. A previous study with MTY‐loaded HEK‐293 and HeLa cells found that KCN appears to dissipate the Δ*T*_app_ more than oligomycin [25] as would be expected. Conversely, Terzioglu et al. [9] unequivocally argue oligomycin can induce a greater decrease in the mitochondrial Δ*T*_*app*_, up to an apparent 17°C–19°C, than what was observed with direct ETS inhibition with antimycin A, rotenone, or cyanide. This was also seen with the ratiometric mito‐gTEMP which confirmed a similar, if not equal Δ*T*_app_ [9].

What is striking is these ETS inhibitors (antimycin A, rotenone, CN^−^) have all been shown to be potent inhibitors of thermogenesis in brown fat mitochondria [46, 47, 48] and oligomycin is routinely used to approximate the proton leak contribution to cellular oxygen flux, which needs to be corrected for non‐mitochondrial oxygen consumption typically by addition of antimycin alone or in combination with other direct ETS inhibitors [19]. The conflicting results between [9, 25] aside, how blocking oxidative phosphorylation could plausibly lead to greater dissipation of Δ*T*_*app*_ than blocking ETS flux raises serious challenges on the veracity of these fluorescence‐based data, especially given the role of ATP in the export of intramitochondrial heat. Moreover, the same paper reports that bongkrekic acid, which will block ANT and therefore have a similar functional consequence on mitochondrial respiratory control as oligomycin, if anything elevated the Δ*T*_*app*_ on addition (Fig. 5 in [9]). The diametrical responses to blocking OXPHOS at the level of ATP/ADP transport (a stable or increased Δ*T*_*app*_) or F_0_F_1_ATPase flux (maximum dissipation of Δ*T*_*app*_) raise more concerns on these mito‐gTEMP findings which also seem to take many minutes to stabilize following oligomycin addition, not unlike the issue raised for MTY above.

## 10 MTY Is Structurally Homologous to Rhodamine Compounds Used to Measure Membrane Potential

Our scope in understanding how cells dynamically respond to physiological stressors and cues has changed drastically in the past 15 years because of the development of new chemical sensors and genetically encoded probes that allow measurements of intracellular pathways in real time [49, 50, 51, 52]. Before implementation, new sensors must be verified and optimized to ensure; (i) probes are not sensitive to microenvironment changes and are selective and specific, (ii) measurements are reproducible and reliable, (iii) can be self‐corrected for signal fluctuations, (iv) have reduced background interference, and (v) enable quantitative analyses [52]. For MTY, Terzioglu et al. [9] assessed its selective accumulation in mitochondria by co‐staining iMEF cells with Mitotracker Deep Red and found that both the MTY and Deep Red signals almost perfectly overlap with one another. Similar findings were made by Chrétien et al. [2] but with HEK293 cells and Mitotracker Green. In addition, it was reported that the MTY dye does not respond to pH, CaCl_2_, or H_2_O_2_ [2, 9]. These findings, coupled with the assumption MTY fluorescence selectively and specifically responds to changes in temperature, led the authors to conclude mitochondria operate at 10°C–12°C hotter than the rest of the cell, sometimes approaching 52°C. Although it is likely there is some component of MTY that is reporting on temperature, in our view the chemical has several caveats that limit the accuracy of this reporting. First, the MTY probe is not ratiometric, which means signal changes in fluorescence are influenced by microenvironment changes and interference from background reactions (e.g., limited by specificity and sensitivity) and cannot be self‐corrected for signal fluctuations. These factors lower the reproducibility and reliability of MTY and its ability to provide accurate quantitative data on temperature changes (if MTY truly is a nanothermometer). Second, the MTY probe is structurally analogous to Mitotracker Red, rhodamine 123, and safranin *O*, dyes used for the fluorescence measurement of ΔΨ_M_ [25]. Mitochondrial MTY uptake is ΔΨ_M_ dependent and therefore it is feasible membrane potential collapse could release accumulated probe from mitochondria (Figure 2B). It is also reasonable to assume dysfunctional mitochondria cannot take up the MTY probe as much as mitochondria with full bioenergetic competency. Another important consideration is the response of MTY to important factors that influence bioenergetics and redox homeodynamics was not rigorously tested. For instance, it was not determined whether changes in MTY fluorescence are sensitive to changes in the ΔΨ_M_ or ΔpH of mitochondria. In this case, experiments with isolated mitochondria wherein fluorescent changes in MTY are compared to safranin *O* under the different states of respiration and in the absence or presence of nigericin should be done. Finally, another crucial aspect is MTY binds to mitochondrial aldehyde dehydrogenase 2 (ALDH2) [24]. ALDH2 uses catalytic thiols to detoxify reactive aldehydes like 4‐hydroxynonenal. Importantly, the MTY and the related chemical, Mitothermo X (MTX) contain a chloroacetyl moiety that is reactive towards thiols [25], raising the question: is it possible the change in MTY fluorescence is due to binding intramitochondrial thiols? Collectively, in our view, the MTY may be reporting on temperature changes but is also likely detecting other intramitochondrial or temporal changes, lending further support to the contention that any conclusion mammalian mitochondria operate at 52°C must be revisited.

## 11 The Physiological Relevance of the “Hot Mitochondria” Hypothesis

The finding by Terzioglu et al. and Chetien et al. that mitochondria could operate at 52°C has been debated by others because the thermal gradient between the mitochondria and the rest of the cell is so extreme that it cannot be explained by the classical laws of physics (the10^5^ gap, which was discussed above). However, as discussed above, elsewhere, and below, the concept of mitochondria operating above 50°C in mammalian cells is also not compatible with our current understanding of cell biology and physiology and the operation of cell stress coping signaling pathways. In most Eutherian mammals, the internal body temperature is generally quite stable and maintained at approximately 37°C, although the set point can vary by species and within a species, not undergoing torpor, by several °C in response to circadian rhythms, infection, cold‐exposure, or exercise [53]. In more extreme cases, mammalian core body temperature can exceed 40°C, like during a heat stroke, which can be fatal because of heat‐induced organ failure [54]. The extreme thermal sensitivity of mammalian cells to heat stress is because an additional 1°C increase in temperature has enough free energy to release the non‐covalent bonds that are necessary to maintain protein and RNA structural stability which, by association, impacts cellular functions [55]. Temperature‐induced protein denaturation occurs for some proteins at temperatures as low as 38°C and can increase drastically when mammalian cells reach 40°C, leading to protein misfolding and aggregation causing proteotoxic stress and the disruption of cellular processes [56]. Some proteins do have higher melting temperatures, such as in the mitochondrial environment, which was recently reported by Jarzab et al., who characterized the “meltome” of 48 000 proteins across 13 species ranging from archaea to humans using a temperature range of 30°C–90°C [57]. Although it is worth noting Chrétien et al. [2] found complex I was highly unstable at temperatures below 45°C and far lower than respiratory complexes II‐IV. In their analysis, Jarzab et al. [57] argue intact mammalian cells are more sensitive to declining respiratory flux with increasing temperature than mitochondria supplied with exogenous substrates within permeabilized cells. We recommend caution on this interpretation because, given the apparent greater thermal lability of complex I [2], it is not clear if the results of Jarzab et al. [57] show differential thermal stability of mitochondria relative to glycolytic enzymes, as claimed, or if the unphysiologically high levels of succinate available to the mitochondria in permeabilized cells circumvented complex I failure while mitochondria in the intact cells were forced to be more reliant on complex I for ETS flux. However, even if some proteins in a mammalian cell melt at higher temperatures, this does not negate the fact that cells have stress coping strategies that are invoked when there is a 1°C increase in the temperature of the cell. To cope with the extreme sensitivity of RNA and proteins to heat‐induced denaturation, mammalian systems have evolved thermosensitive protein sensors, like heat shock factor‐1 (HSF1) and heat shock protein 70 (HSP70) that allow the induction of adaptive stress responses to slight increases in temperature. HSF1 and HSP70 are responsible for orchestrating the heat shock response (HSR), which cross‐communicates with other important stress response pathways like unfolded protein response (UPR), electrophilic stress responses (ESR), DNA damage response (DDR), mitochondrial stress responses (MSR), and integrated stress responses (ISR) to prevent proteotoxicity, calcium imbalances, and oxidative stress and damage [58, 59, 60]. Collectively, these pathways (HSR, UPR, ESR, DDR, MSR, ISR) work together to invoke cell stress coping strategies to correct protein misfolding, restore redox homeodynamics, repair DNA damage, and rebalance calcium homeostasis in response to cell stress [61]. Essentially, these pathways are needed to restore homeostasis or promote allostasis in response to the various stresses that are faced by mammalian cells, like a small change in temperature. If a stress like a slightly higher temperature is constant and cell homeostasis cannot be restored, then the cell commits to cell death.

The observation that mitochondria could operate at 10°C–12°C above 37°C or higher (up to 52°C as measured with MTY) is difficult to reconcile because it is incompatible with the function of cell stress sensing. Essentially, if mitochondria are operating at such high temperatures, then it would be anticipated that the HSR, UPR, ESR, DDR, MSR, and ISR are constantly being activated. In fact, as described above, the inability of cells to resolve a heat stress (or other stresses) inevitably leads to the activation of cell death pathways. It was posited that the ability of mitochondria to operate at such high temperatures is due to the greater resistance of the organelle to thermal stress, which could be due to the presence of thermal protectant solutes and higher levels of heat shock proteins and purine tracks in mitochondrial DNA [26, 62]. However, these factors would not be enough to negate proteotoxicity and the induction of cell death in response to a persistent thermal stress. The recent characterization of the cell meltome supports this notion because the mitochondrial electron transport chain (ETC) enzymes were found to have a melting temperature of ~46°C, with T cells approaching a melting temperature of ~60°C [57]. However, as noted above, Chrétien et al. found complex I is highly unstable at 45°C, which means complex I driven respiration is compromised at 45°C even if the average thermal stability of the ETC is beyond this temperature. Besides, even if all mitochondrial components could tolerate temperatures beyond 45°C, it does not change the fact that these conditions would activate the cell stress coping strategies listed above and even cause cell death. In addition, the physiological relevance of the finding that mitochondria could tolerate more thermal stress was diminished by the observation in the same study that the respiratory rate of intact cells, which rely on glycolytic enzymes to provide carbon fuel for the mitochondrial ETS, rapidly decreased at a temperature of 42°C [57]. This shows thermal stress compromises the capacity of mammalian cells to use a critical fuel like glucose for energy metabolism. In addition, the cell meltome work reported by Jarzab et al. is inconsistent with several studies showing that incubating isolated liver mitochondria and several cultured cell lines at temperatures > 43°C degrades the respiratory complexes and supercomplexes [8] and compromises mitochondrial membrane fluidity, increases ROS production, and depolarizes the ΔΨ_M_, leading to the induction of cell death [62, 63, 64, 65, 66, 67]. Based on these findings, it is reasonable to conclude that “hot mitochondria” would be constantly triggering cell stress responses and cell death pathways. Another important consideration is that advances in molecular biology techniques have revealed that stress detecting proteins in cells are strategically positioned in subcellular locations to allow for the rapid sensing of stresses like temperature changes. For example, cell stress coping sensor proteins localize to the surface of mitochondria, including HSF1 [68, 69]. HSF1 is activated when it is released by HSP70 in response to unfolded proteins. This promotes HSF1 localization to the nucleus for the activation of genes involved in resolving thermal stress. It has been found that mitochondria‐localized HSF1 is needed for resolving mitochondrial proteotoxicity and that over‐stimulation of this pathway is related to Huntington's Disease [68, 69]. Thus, even if mitochondria are more tolerant to thermal stress and it is anticipated that the thermal gradient dissipates too quickly to warm adjacent organelles and the cytosol, it is likely that cell stress sensors located on the surface of mitochondria, like HSF1, are activated by the high temperatures. This would result in the induction of the cell stress coping responses listed above and, if the thermal stress persists, cell death.

## 12 Conclusion

The fluorescence data from MTY, and compounds like mito‐gTEMP, will have some component of local temperature within them, but the extraordinarily slow responses of these compounds appear incompatible with the expected rapid conductive movement of heat combined with the kinetics of mitochondrial heat production (Figure 3). Furthermore, these fluorescent probes provide responses to mitochondrial inhibitors inconsistent with the expected dynamics of heat liberation from substrate oxidation (Figure 4). While there are several alternative studies that indicate a pulse of heat release with uncoupling of mitochondria [11, 12, 13], suggesting some degree of mitochondrial Δ*T*_*app*_, these thermal gradients appear on time scales much faster than those assessed using MTY and mitochondrial cooling. Additionally, there are other examples of chemical uncoupler induced evidence for a mitochondrial Δ*T*_*app*_ much smaller than that argued for by using MTY, leaving the possibility that some, smaller, intracellular thermal gradients may occur.

In conclusion, the extreme mitochondrial Δ*T*_*app*_ values of > 10°C that have been used to support the ‘hot mitochondrial’ concept [2, 9], heavily based on apparent cooling of ‘hot mitochondria’ following some inhibition of ETS flux, fail to give responses that are consistent with these compounds acting as nanothermometers that are uniquely reporting temperature and solely temperature. Given the membrane potential‐dependent uptake of MTY and similar compounds, we speculate that the slow phase of the responses may reflect redistribution of the fluorophores, possibly as part of the energy‐independent binding known for other compounds accumulated within mitochondria by membrane potential dependent mechanisms and thus may have little to do with very fine scale resolution of local temperature. Based on this, we recommend rejecting the contention mitochondria normally operate with an extreme Δ*T*_*app*_ of > 10°C.

## Author Contributions

**Ryan J. Mailloux:** conceptualization, writing – original draft, writing – review and editing, formal analysis. **Jason R. Treberg:** conceptualization, formal analysis, writing – original draft, writing – review and editing, data curation.

## Funding

This work was supported by the Natural Sciences and Engineering Research Council of Canada (RGPIN‐2024‐06035, RGPIN‐2022‐03240).

## Disclosure

Permission to Reproduce Material From Other Sources: Reproduced material was originally published under the Creative Commons Attribution 4.0 International Public License (CC BY 4.0; https://creativecommons.org/licenses/by/4.0/).

## Ethics Statement

The authors have nothing to report.

## Consent

The authors have nothing to report.

## Conflicts of Interest

The authors declare no conflicts of interest.

## Data Availability

Data sharing not applicable to this article as no datasets were generated or analysed during the current study.
